# Finite element analysis and clinical application of 3D-printed Ti alloy implant for the reconstruction of mandibular defects

**DOI:** 10.1186/s12903-024-03857-y

**Published:** 2024-01-17

**Authors:** Runqi Xue, Qingguo Lai, Hongyu Xing, Chiyang Zhong, Yixuan Zhao, Kaiwen Zhu, Yanwei Deng, Chengbin Liu

**Affiliations:** 1 Department of Oral and Maxillofacial Surgery, The Second Hospital of Shandong University, Jinan, Shandong Province China; 2 Shandong University, Jinan, Shandong Province China; 3 School of Mechanical and Electronic Engineering, Shandong Jinan University, Jinan, Shandong Province China; 4 Zaozhuang Municipal Hospital, Zaozhuang, Shandong Province China

**Keywords:** 3D printing, Maxillofacial surgery, Reconstruction, Finite element analysis, Biomechanics, Personalized medicine

## Abstract

**Background:**

The reconstruction of segmental defect of the mandible has always been a challenge. The customized reconstruction plate without a bone graft is also considered a transitional means of rehabilitation and reconstruction in some cases.

**Methods:**

This study evaluated the biomechanical behaviors of customized plates with different structural designs comparing with commercial plates using the finite element method in reconstrution of the lateral mandible defect.

**Results:**

Simulations revealed the stress state in the plate bodies, bone tissues and screws were associated with the width, height, thickness of the plates as well as the distribution of screws. In all of the groups, the system of 16 mm-high, 2.8 mm-thick customized reconstruction plate with 10 screws was considered to be the most ideal design because of the most harmonious biomechanical state. What’s more, the stress shielding effects were not obvious in this experiment. Based on the above findings, we conducted a clinical case analysis to verify the mechanical properties of customized reconstruction and obtained a satisfactory operation result.

**Conclusions:**

The results show that by adjusting the contour parameters of the reconstruction plates, an ideal and reliable customized plate can be manufactured. And the customized 3D-printed Ti alloy implant will be a new way to achieve mandibular reconstruction in patients unable to perform autologous bone graft surgery.

**Trial registration:**

The present trial has been registered with ChiCTR, the registration number is ChiCTR 2,000,038,973 on 11/10/2020.

**Supplementary Information:**

The online version contains supplementary material available at 10.1186/s12903-024-03857-y.

## Introduction

The mandible is the only load-bearing bone of craniomaxillofacial bones whose integrity is vital for facial esthetic and stomatognathic function [1]. The simultaneous or delayed reconstructions of segmental mandibular defects caused by tumors, trauma, inflammation, and congenital development malformation are significant challenges in oral and maxillofacial surgery. According to the statistics of the causes of maxillofacial defects in Affiliated 9th People’s Hospital, Shanghai Jiaotong University, mandibular defects accounted for 17.38% [2]. Autogenous bone graft is presently considered as the ‘’gold standard’’ for the mandibular reconstruction [3]. This generally acknowledged method is limited in cases with insufficient bone mass and highly malignant carcinoma [4]. A common site for autologous bone extraction of vascularized autologous bone graft include an iliac crest and fibula, etc. In many cases, patients cannot undergo autologous bone extraction surgery, such as advanced age unable to tolerate prolonged general anesthesia surgery, osteoporosis, history of lower limb fracture or loss, and developing children. Insufficient autologous bone mass means that the bone mass in the donor area is less than that required in the recipient area. Highly malignant tumors indicate a high degree of malignancy, rapid growth of tumor cells, and easy-to-occur regional lymph node metastasis and distant metastasis. The chance of surgical resection is often lost when it is found, or even if surgical resection is performed, the risk of postoperative recurrence and metastasis is high, so adjuvant radiotherapy or chemotherapy is often needed as soon as possible after surgery. In that case, simple prosthesis reconstruction may serve as an alternative. However, neither prostheses nor vascularized flaps can match the mandibular growth and development in children. It is reported that spontaneous bone regeneration (SBR) which is defined as the unexpected formation of new cortical bone can occur in mandibular resection surgeries with or without bone graft, and the osteogenesis is strongly influenced by age [5]. In infants and small children, the immediate reconstruction of bone grafting may be delayed to allow for spontaneous regeneration of the mandible at the defect site [6]. However, Spontaneous bone regeneration following the loss of the mandible has occasionally been reported in the literature. Long-term follow-up of the regenerated mandible is rare in the literature. Therefore, some studies have pointed out that the reconstruction plate alone is a desirable transitional method [7].

A large number of clinical retrospective studies have shown that reconstruction plates alone are accompanied by various complications, including exposure, fracture and screw loosening [8]. The main factors are as follows: stress fatigue caused by repeatedly pre-bending, lacking passive anatomical fitting and stress shielding effect, etc. [9, 10]. From the biomechanical point of view, the existence of a reconstruction plate will protect the bone tissue with stress shielding for the fracture healing while producing stress-shielding effect on the surrounding bone tissue, leading to the change of the normal stress-strain microenvironment of the original bone tissue and bone resorption or even bone injury owing to the gap between the elastic modulus and the overall strength of the plate. By adjusting the fit of the plates, introducing adjustable porosity and lightweight structure design, the stress shielding effect can be reduced [11]. 3D-printed customized reconstruction plate is put forward because of the computer-aided design of customized shape and optimized structure, which can solve the above problems [12] and be beneficial to the patients better suited to the method of reconstruction plate alone. Its reduced stress shielding effect and accurate anatomical fitting may promote the SBR in children. This is a leap forward from the current reliance on commercially available plates which will change the current mandibular defect reconstruction strategy of treatment to minimize trauma and to promote personalized therapy [13].

Segmental mandibular defects can be divided into eight types by (HCL) classification [14]. A retrospective observational study demonstrates that the lateral defects show the lowest complications (51.4%) and a high success rate of reconstruction plate [15]. Considering that the bite force borne by the reconstruction plate will increase with the development of the mandible, this study used a lateral defect of an adult mandibular model to evaluate the biomechanical properties of 3D-printed customized titanium plates with different parameters in comparison to commercial plates by finite element analysis and observe its feasibility through case analysis.

## Materials and methods

### Segmental mandibular defect 3D model preparation

An adult patient with a symmetric mandible without fracture, tumor, and inflammation was required. A 64-row spiral computer tomography (CT) was used to scan the patient’s skull with three-dimensional (3D) reconstruction, with a slice thickness of 0.625 mm, and stored in a disc as digital imaging and communication in medicine (DICOM) format for further study. Stereolithographic (STL) mandibular model composed of cortical bone, cancellous bone, tooth, and joint fossa were fabricated using the patient’s CT image data and Mimics Medical 21.0 (Materialise, Belgium) software according to the thresholding value of Hounsfield unit (Hu). The threshold is usually set at 900–3000 Hu, which can effectively discard the soft tissue influence and retain a relatively complete mandibular image. The STL files were subsequently imported into Geomagic Wrap 2017 (3D Systems, America) software to repair surface mesh and automatically trim the surface, then converted into x_t format. The x_t files of cortical bone, cancellous bone, tooth were imported into NX 12.0 (Siemens, Germany) software for Boolean operation to ensure that the models were in close contact and did not overlap each other. NX 12.0 software could automatically identify and assemble each part of the model into a complete mandibular model. The mandible model including bilateral joint fossa was saved in both x_t and STL format. Then, the mandible was segmented from the proximal surface of the first premolar to the distal surface of the second molar of the right jaw by Boolean operation, and the remaining mandibular bone was saved in x_t format. The 3D model of the mandibular segmental defect was obtained with the a defect length of more than 4 cm, which exceeded the critical mandibular defect.

### Design of customized reconstruction plates, commercial reconstruction plate, and screws

In this study, the design of the commercial reconstruction plate and screws referred to AO MATRIXMANDIBLE reconstruction plate, was straight, with 20 holes and self-locking screws, and were simplified. A smooth curve was drawn on the outer layer of the right jaw, above the inferior margin about 5 mm of the mandibular model, to match the patient’s jaw contours. The total length and thickness of the commercial reconstruction plate are 110 mm and 2.5 mm, including 14 retention screw holes which were evenly distributed with 8 mm hole spacing.

The body of the screw was simplified to a cylinder and cone without thread structure, and the diameter of the cylinder was 2.4 mm while the length was 12 mm, and the bottom diameter of the cone was 2.4 mm while the half angle was 59°. The head of the screw was designed as a cone with a bottom diameter of 2.4 mm, a top diameter of 3.5 mm, a height of 1.2 mm. If the screw penetrated the lingual cortical bone, the length of the cylinder would be adjusted to 10 mm.

The extracted mandible STL file was imported into Geomagic Design X 2016.2.1 (3D Systems, America) software, and a non-uniform rational basis splines (NURBs) from the posterior edge of the right mandibular ramus to the left mandibular incisor, whose upper edge was parallel to the junction of alveolar process and the body of mandible and lower edge was parallel to the inferior margin of mandible, was saved as an x_t format to obtain the curved surface of customized reconstruction plates. The mandibular segmental defect 3D model and the customized reconstruction plate curved surface were assembled in NX software to design the customized reconstruction plates which were saved in x_t format. The main differences between the plates were in height, thickness, and the number of fixing screws. The length of the plates included the length of the defect and the proximal and distal extensions whose lengths were 26 mm ensuring that at least 3 screws can be nailed. The height of customized reconstruction plates were 12 mm, 16 mm, and 20 mm, and the thickness were 2.0 mm, 2.4 mm, and 2.8 mm, respectively, a total of 9 kinds of customized reconstruction plates. Taper screw holes were generated in the plates with the shape of countersink, whose diameters were 3.5 mm and 2.4 mm with an angle of 45°. One row of 5 screw holes was reserved in the defect area of the 12 mm reconstruction plate, and 2 rows of 4 screw holes were reserved in the proximal and distal extension plate with 4 screws being nailed into. As for the 16 and 20 mm reconstruction plates, the reserved screw holes in the defect area were 2 rows of 10 and 3 rows of 15, and in the extensional part were 2 rows of 7 screw holes with 5 screws being nailed into, and 3 rows of 9 screw holes with 6 screws.

### Finite element analysis

For the existence of a very complex combination of factors, such as non-uniform material properties, arbitrary boundary conditions, complex geometric shapes, and others mixed, the finite element method can be flexible to deal with and solve, which is exactly what the reconstructed mandibular looks like under different forces. To simulate the biomechanical response on the reconstruction plates and bones, a finite element model of a mandibular framework consisting of cortical bone, cancellous bone, tooth, and joint fossa were imported into Workbench 14.5 (ANSYS, America).

The material properties of bone and tooth were characterized as homogeneous and isotropic and were obtained from literature studies. A homogeneous isotropic linearly elastic model was used to define the reconstruction plates and fixing screws made from titanium (Ti-6Al-4 V). The material properties used in the finite element (FE) model are referred to other literature in Table 1.

**Table 1:** Young’s modulus and poisson’s ratio of cortical bone, cancellous bone, tooth and titanium alloy

	Young modulus/MPa	Poisson’s ratio
cortical bone	13,700	0.3
cancellous bone	1850	0.3
tooth	20,000	0.3
titanium (Ti-6Al-4 V)	113,800	0.342

To reduce the computation time and to avoid geometrical errors, the cortical and cancellous bone were meshed by the Automatic mesh generating method, the tooth, and bilateral joint fossa were meshed by the Tetrahedral patch independent method, the reconstruction plates and screws were meshed using Hex dominant method, and the quality of meshing was adjusted by Global setting and local sizing design to ensure the Orthogonal Quality of Mesh Metric was about 0.8.

To simulate the joint movement when chewing, Fixed-Joints were defined between the joint fossa and condylar process, in which the joint fossa was the reference body while the condylar process was the mobile body. Meanwhile, displacement in all directions was restricted in the lateral joint fossa. Boned connections were defined between all remaining interfaces. Considering the actual occlusion situation of the patient with mandibular segmental defect, two kinds of occlusion, incisal clenching (INC) and left molar clenching (MOL-L) were selected based on the literature and the discussion with oral and maxillofacial surgeons. The loads setting of the FE model consisted of chewing force and force of masticatory muscles which included masseter (shallow masseter, SM; deep masseter, DM), temporal muscle (anterior temporal muscle, AT; medial temporal muscle, MT; posterior temporal muscle, PT), medial pterygoid (MP) and lateral pterygoid (superior lateral pterygoid, SLP; inferior lateral pterygoid, ILP). Values and directions of individual muscle forces and chewing force were derived from literature as Table 2 [16] and Table 3 [17].

**Table 2:** The strength of each masticatory muscle under INC and MOL-L

	INC						MOL-L					
	R			L			R			L		
(/N)	x	y	z	x	y	z	x	y	z	x	y	z
SM	-15.8	-57.7	31.9	15.8	-57.7	31.9	-23.6	-101	47.9	28.4	-121.2	57.4
DM	-11.6	-16.1	7.6	11.6	-16.1	7.6	-26.7	-37.1	17.5	32.1	-44.5	21
PM	66.3	-107.8	50.9	-66.3	-107.8	50.9	71.4	-116	54.8	-71.4	-116.1	54.8
AT	-1.9	12.5	0.6	1.9	12.5	0.6	-13.7	90.5	4.0	17.2	114	5.1
MT	-1.3	4.8	2.9	1.3	4.8	2.9	-14.2	53.6	32	14	52.8	31.5
PT	-0.6	1.4	2.6	0.6	1.4	2.6	-6.1	14	25.2	9.3	21.1	38.1
ILP	29.9	-8.3	36	-29.9	-8.3	36	27.4	-7.6	33	-12.6	-3.5	15.2
SLP	10.9	1.1	9.3	-10.9	1.1	9.3	0	0	0	0	0	0

**Table 3:** The occlusal force of each teeth under INC and MOL-L

	33	32	31	41	42	43	44	45	46	47
INC/N	100	60	60	60	60	100	0	0	0	0
MOL-L/N	0	0	0	0	0	0	120	150	200	200

### Clinical applications

**Fig. 1:**
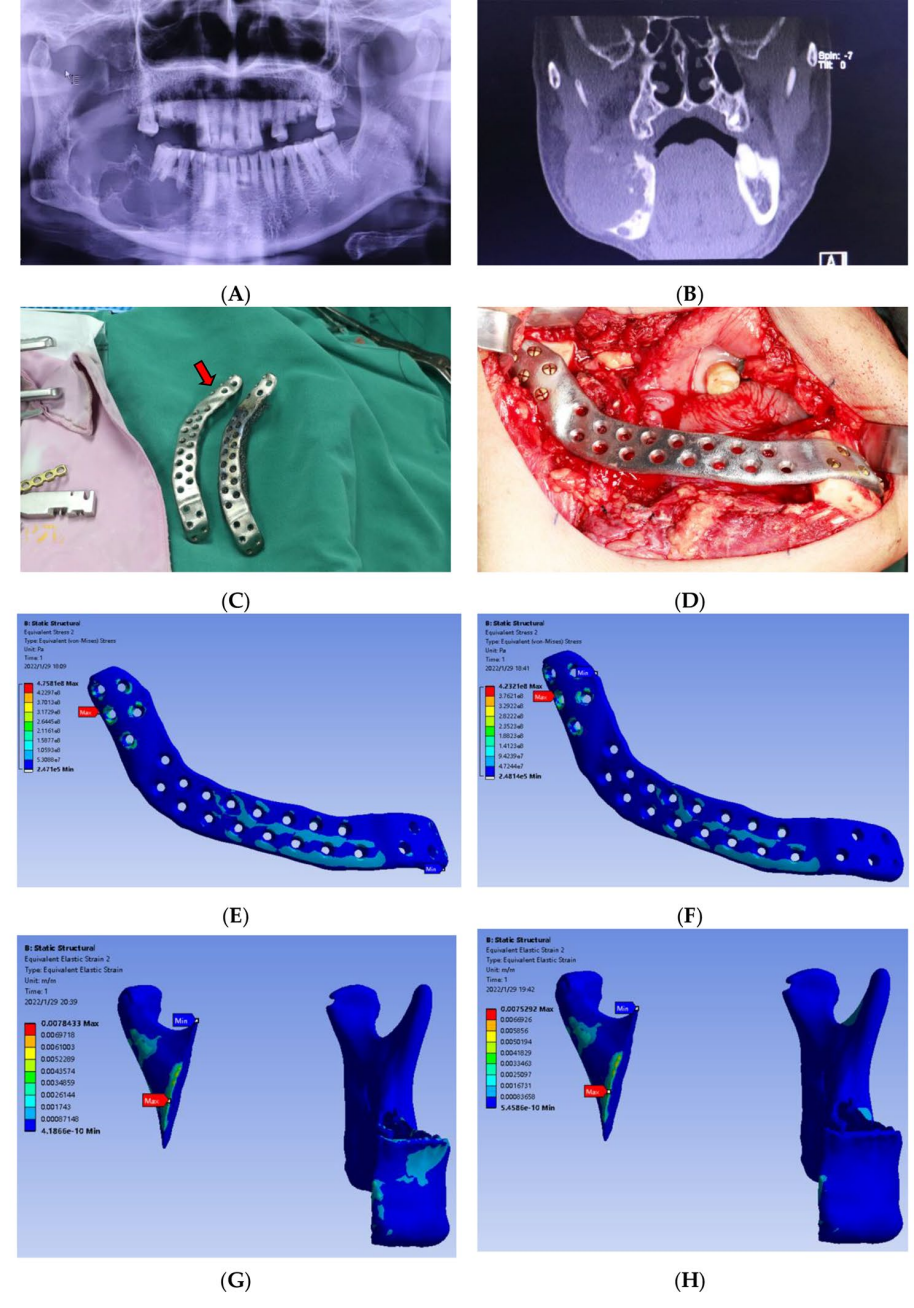
An adult clinical application case of 3D printing customized reconstruction plate and its finite element analysis: (**A**) The radiographic results of surface tomography shown an occupying lesion with a range of 5 cm×4 cm×4 cm in the right mandible; (**B**) The radiographic results of computed tomography examination shown the soft tissue intrusion; (**C**) The red arrow shown the 3D-printed customized reconstruction plate which was 16 mm in width and 2.8 mm in thickness; (**D**) Intraoperative photographs; (**E**) The Equivalent Stress distributing on the 3D printing customized reconstruction plate in INC; (**F**) The Equivalent Stress distributing on the 3D printing customized reconstruction plate in L-MOL; (**G**) The Equivalent Strain distributing on the bone defect area in the internal fixation areas on the outside in INC; (**H**) The Equivalent Strain distributing on the bone defect area in the internal fixation areas on the outside in L-MOL.

A 78-year-old male was diagnosed with ameloblastoma in the right mandible. The tumor recrudesced five years after curettage. The affected mandible was swelling, and the imaging examination indicated that the right mandible had an occupying lesion with a range of 5 cm×4 cm×4 cm and was accompanied by soft tissue intrusion(Fig. 1A and B). All the feasible options for mandibular reconstruction were explained to the patient and his family, including vascularized autogenous bone graft, autogenous bone graft, commercial reconstruction plate, and customized reconstruction plate. The advantages and disadvantages of each method were fully explained and they finally chose the customized reconstruction plate. Considering the old age and weak body condition, we choose a simple reconstruction plate to repair the continuity of the partly removed mandible after informed consent (Fig. 1C).

The 3D-printed customized reconstruction plate was 16 mm in width and 2.8 mm in thickness, with 5 screw holes in the proximal and distal extensions(Fig. 1D). We performed the finite element analysis to verify its mechanical properties. This case analyzed whether there was a stress concentration point, whether the maximum stress was over the maximum elastic modulus, and whether the bone tissue under the reconstruction plate suffered the stress shielding effect under the condition of the real bite force in the human body. In turn, we can verify the accuracy of the finite element analysis method, and predict the possible complications, such as titanium plate fracture, screw loosening, etc.

In addition, we plan to conduct follow-up examinations with CT every 2 months for the first 6 months and then every year for the long-term follow-up of the in vivo changes of the customized reconstruction plate and the recurrence.

## Results

The finite element evaluation of reconstruction plates and bone tissues covered by plates focused on the biomechanical properties including the von-mises stress and equivalent strains distributing on the segmental mandibular defect model with various reconstruction plates, and the axial deformations of screws under different occlusion situations.

### Biomechanical analysis of reconstruction plates

Compared to the tensile yield strength (880 MPa) of Ti-6Al-4 V, the maximum von-mises stress of commercial reconstruction plates and 12 mm-high customized reconstruction plates were significantly higher than the limit, while the 16 mm-high and 20 mm-high customized reconstruction plates remained safe as seen in Table 4.

**Table 4:** The maximum von mises stresses of commercial reconstruction plates and customized reconstruction plates under INC and MOL-L

the maximum von mises stresses(/MPa)	commercial reconstruction plate	customized reconstruction plates								
		12 mm			16 mm			20 mm		
		2.0 mm	2.4 mm	2.8 mm	2.0 mm	2.4 mm	2.8 mm	2.0 mm	2.4 mm	2.8 mm
INC	907.16	1113.8	1358.3	971.48	679.69	802.98	635.71	442.54	761.38	543.55
MOL-L	1006.1	990.15	1193.1	863.7	626.01	710.36	546.21	410.91	696.38	478.48

**Fig. 2:**
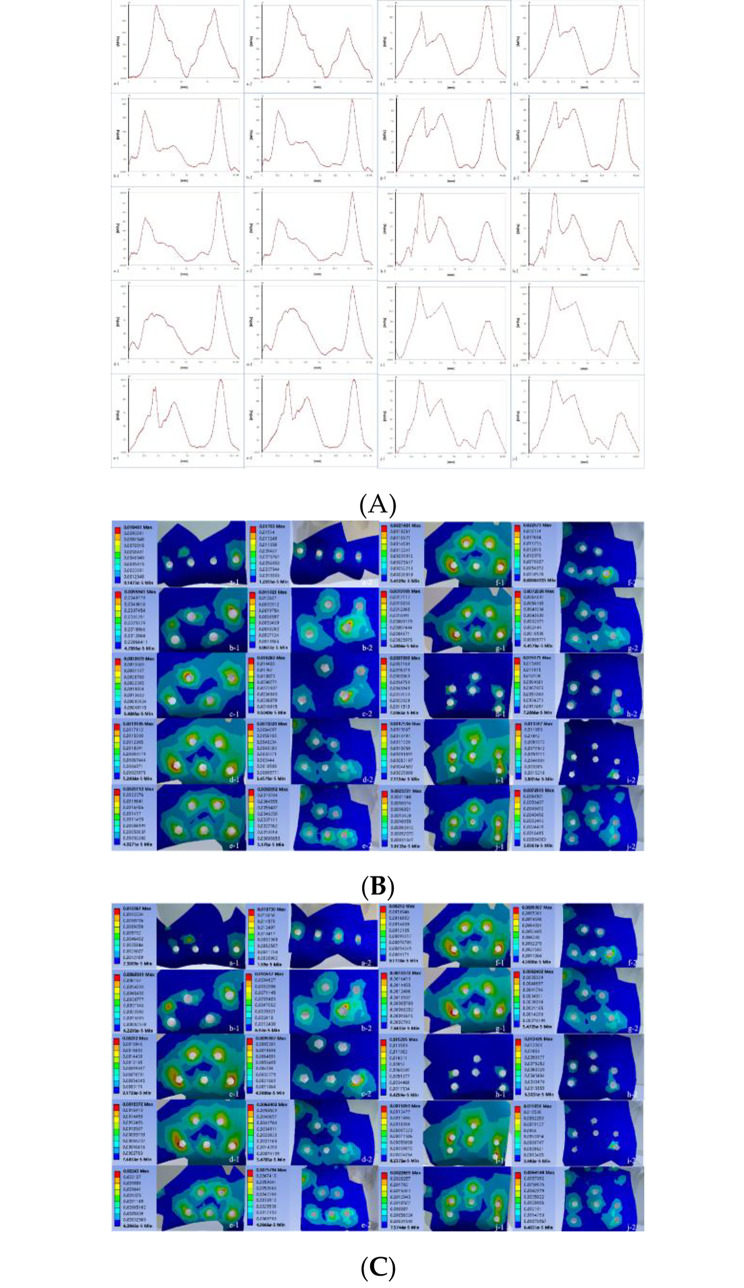
Biomechanical analysis of reconstruction plates and the bone tissue covered by reconstruction plates. (**A**) The variation tendency of von-mises stress distributing on the edge of customized reconstruction plates and commercial reconstruction plates: (a-1)-(j-1) distal parts of plates; (a-2)-(j-2) mesial parts of plates; (a) commercial reconstruction plates; (b) 12 mm-height, 2.0 mm-thickness customized reconstruction plates; (c) 12 mm-height, 2.4 mm-thickness customized reconstruction plates; (d) 12 mm-height, 2.8 mm-thickness customized reconstruction plates; (e) 16 mm-height, 2.0 mm-thickness customized reconstruction plates; (f) 16 mm-height, 2.4 mm-thickness customized reconstruction plates; (g) 16 mm-height, 2.8 mm-thickness customized reconstruction plates; (h) 20 mm-height, 2.0 mm-thickness customized reconstruction plates; (i) 20 mm-height, 2.4 mm-thickness customized reconstruction plates; (j) 20 mm-height, 2.8 mm-thickness customized reconstruction plates. (**B**) The minimum and maximum relative strains of bone tissues distributing in the bone-plate contact areas under INC: (a-1)-(j-1) distal parts of bone-plate contact areas; (a-2)-(j-2) mesial parts of bone-plate contact areas; (a) commercial reconstruction plates; (b) 12 mm-height, 2.0 mm-thickness customized reconstruction plates; (c) 12 mm-height, 2.4 mm-thickness customized reconstruction plates; (d) 12 mm-height, 2.8 mm-thickness customized reconstruction plates; (e) 16 mm-height, 2.0 mm-thickness customized reconstruction plates; (f) 16 mm-height, 2.4 mm-thickness customized reconstruction plates; (g) 16 mm-height, 2.8 mm-thickness customized reconstruction plates; (h) 20 mm-height, 2.0 mm-thickness customized reconstruction plates; (i) 20 mm-height, 2.4 mm-thickness customized reconstruction plates; (j) 20 mm-height, 2.8 mm-thickness customized reconstruction plates. (**C**) The minimum and maximum relative strains of bone tissues distributing in the bone-plate contact areas under MOL-L: (a-1)-(j-1) distal parts of bone-plate contact areas; (a-2)-(j-2) mesial parts of bone-plate contact areas; (a) commercial reconstruction plates; (b) 12 mm-height, 2.0 mm-thickness customized reconstruction plates; (c) 12 mm-height, 2.4 mm-thickness customized reconstruction plates; (d) 12 mm-height, 2.8 mm-thickness customized reconstruction plates; (e) 16 mm-height, 2.0 mm-thickness customized reconstruction plates; (f) 16 mm-height, 2.4 mm-thickness customized reconstruction plates; (g) 16 mm-height, 2.8 mm-thickness customized reconstruction plates; (h) 20 mm-height, 2.0 mm-thickness customized reconstruction plates; (i) 20 mm-height, 2.4 mm-thickness customized reconstruction plates; (j) 20 mm-height, 2.8 mm-thickness customized reconstruction plates

As seen in Fig. 2A, the maximum von-mises stress distribution on the edge of customized reconstruction plates and commercial reconstruction plates were similar. The closer to the bone defect boundary, the greater the stress on the plate was. Under the incisal clenching, the maximum von Mises stress on the commercial reconstruction plate was 312.6 MPa (Fig. 2A a-1). Under the left molar clenching, the maximum von Mises stress on the commercial reconstruction plate was 356. 1 MPa (Fig. 2A a-2). Under the incisal clenching, the maximum von Mises stress on 12 mm-high customized reconstruction plates was 213.5 MPa (2.0 mm-thickness) (Fig. 2A b-1). Under the left molar clenching, the maximum von Mises stress on 12 mm-high customized reconstruction plates was 222.4 MPa (2.0 mm-thickness) (Fig. 2A b-2). The von Mises stress curve of the 20 mm-high customized reconstruction plates under the two occlusal conditions was more “gentle” than that of other groups, indicating that the stress distribution on the reconstruction plates was more symmetrical than that of other groups.

In clinical applications, to prevent the occurrence of complications of plate fractures, the structure of the plate should be devoid of stress concentration points, and the maximum stress should not exceed the material’s elastic modulus. Furthermore, the more uniform the distribution of stress across the titanium plate, the better we consider its long-term performance. At the same time, if the fixability of the titanium plate is guaranteed, the smaller the stress on the titanium plate, the mechanical stimulation put on the bone tissue is greater, thereby reducing the stress shielding effect produced by the plates.

### Biomechanical analysis of bone tissue covered by reconstruction plates

To study the biomechanical properties of the bone tissue covered by a titanium plate, the four mechanical usage windows defined by mechanostat theory, including disuse window, physiological window, overuse window and pathological overload window, were introduced in this paper [18]. The local deformation imposed by mechanical loads in the tissue is called strain (1 µstrain equals 1 μm deformation per meter, 1 μm/m = 10^− 6^ mm/mm). It is considered that both bone resorption and bone degeneration will cause the loose of reconstruction plates.

The results of the minimum and maximum relative strains of bone tissues distributing in the bone-plate contact areas, which were closely related to post-operative failures under different occlussion (INC and MOL-L), were shown in Fig. 2B and C; Table 5. According to the strain distribution of bone tissues, even if the maximum strain value was located in the pathological overload window or the minimum strain value was located in the disuse window, most of the bone tissues were still in a normal mechanical environment except for some points.

**Table 5:** Biomechanical analysis of bone tissue covered by reconstruction plates: the relative strains of bone tissues in bone-plate contact areas under INC and MOL-L

the relative strains of bone tissues		commercial reconstruction plates	customized reconstruction plates								
			12 mm			16 mm			20 mm		
			2.0 mm	2.4 mm	2.8 mm	2.0 mm	2.4 mm	2.8 mm	2.0 mm	2.4 mm	2.8 mm
mesial bone-Plate contact areas (INC)	Min	1.3 × 10^− 5^	8.1 × 10^− 5^	9.5 × 10^− 5^	6.5 × 10^− 5^	5.2 × 10^− 5^	6.2 × 10^− 4^	6.5 × 10^− 5^	7.2 × 10^− 5^	3.9 × 10^− 5^	3.9 × 10^− 5^
	Max	1.7 × 10^− 2^	1.2 × 10^− 2^	1.6 × 10^− 2^	7.2 × 10^− 3^	8.3 × 10^− 3^	2.3 × 10^− 2^	7.2 × 10^− 3^	1.5 × 10^− 2^	1.3 × 10^− 2^	7.3 × 10^− 3^
distal bone-plate contact areas(INC)	Min	8.1 × 10^− 5^	4.8 × 10^− 5^	5.4 × 10^− 5^	5.2 × 10^− 5^	4.9 × 10^− 5^	5.4 × 10^− 5^	5.2 × 10^− 5^	7.1 × 10^− 5^	7.1 × 10^− 5^	5.6 × 10^− 5^
	Max	1.1 × 10^− 2^	5.6 × 10^− 3^	4.0 × 10^− 3^	1.2 × 10^− 3^	2.5 × 10^− 3^	2.2 × 10^− 3^	1.9 × 10^− 3^	9.8 × 10^− 3^	1.8 × 10^− 3^	2.4 × 10^− 3^
mesial bone-Plate contact areas (MOL-L)	Min	1.6 × 10^− 5^	7.0 × 10^− 5^	4.6 × 10^− 5^	5.5 × 10^− 5^	4.1 × 10^− 5^	4.6 × 10^− 5^	5.5 × 10^− 5^	6.3 × 10^− 5^	3.7 × 10^− 5^	6.4 × 10^− 5^
	Max	1.9 × 10^− 2^	1.1 × 10^− 2^	9.6 × 10^− 3^	6.2 × 10^− 3^	7.6 × 10^− 3^	9.6 × 10^− 3^	6.2 × 10^− 3^	1.3 × 10^− 2^	1.2 × 10^− 2^	6.4 × 10^− 3^
distal bone-plate contact areas(MOL-L)	Min	7.3 × 10^− 5^	6.2 × 10^− 5^	9.2 × 10^− 5^	1.1 × 10^− 4^	6.3 × 10^− 5^	9.2 × 10^− 5^	7.4 × 10^− 5^	6.4 × 10^− 5^	8.2 × 10^− 5^	7.6 × 10^− 5^
	Max	1.0 × 10^− 2^	6.9 × 10^− 3^	2.1 × 10^− 3^	3.0 × 10^− 3^	2.4 × 10^− 3^	2.1 × 10^− 3^	1.8 × 10^− 3^	1.5 × 10^− 2^	1.5 × 10^− 3^	2.3 × 10^− 3^

In this experiment, the effect of stress shielding was not obvious in bone tissues distributed in different parts under the clenching condition of INC and MOL-L. The bone tissues of the bone-plate contact areas were more likely to overload in the distal mandible under both INC and MOL-L in customized reconstruction plates, while the region of stress concentration was near the defect in the commercial reconstruction plates. Analyzing the strain distribution on the bone tissue surface under a reconstruction plate can aid in the precise adjustment of the design of customized reconstruction plates. We propose that the reconstruction plates should be appropriately thickened at sites of bone injury, and suitably thinned at sites of bone resorption. Perhaps, this can be achieved through topological optimization.

### Mechanical analysis of the screws

According to the analysis of the axial deformations of screws under INC and MOL-L (Table 6), there were significant differences in the force on the screws between commercial reconstruction plates and customized reconstruction plates, including the distribution of stress and the location of the maximum relative stress. A negative deformation value meant the screw was pushed along the longitudinal axis toward the inner cortex whilst a positive value indicated a screw was pulled out. In the internal fixation system, the larger the pull-out force received, the higher risk of screw loosening. The bone tissue overload was easy to occur when a greater push force was applied. In the commercial reconstruction plate, the screws retaining in the mesial mandible received more pull-out force than the screws in the distal mandible, and the screws farthest from the defect in the mesial mandible received the most concentrated pull-out force. In the customized reconstruction plates, the screws retaining in the distal mandible received a greater force, while all the screws in the distal mandible were pushed along the longitudinal axis.

**Table 6:** Mechanical analysis of the screws: the axial displacements of screws of commercial reconstrction plates, 12 mm-height reconstrction plates, 16 mm-height reconstrction plates and 20 mm-height reconstrction plates under INC and MOL-L. * ”L” represents the screw fixed to the meial bone tissue. “L1”-”L6” is from bottom to top and from left to right; ”R” represents the screw fixed to the distal bone tissue. “R1”-”R6” is from bottom to top and from left to right

axial displacements of screws(/mm)		L1^*^	L2^*^	L3^*^	L4^*^	L5^*^	L6^*^	R1^*^	R2^*^	R3^*^	R4^*^	R5^*^	R6^*^
Commercial Reconstruction plate	INC	0.323	0.314	0.312	0.318			0.215	0.221	0.230	0.238		
	MOL-L	0.375	0.355	0.353	0.368			0.107	6.3 × 10^− 2^	1.8 × 10^− 2^	-2.9 × 10^− 2^		
12 − 2.0	INC	-2.6 × 10^− 2^	-2.8 × 10^− 2^	-2.5 × 10^− 2^	-2.4 × 10^− 2^			-0.437	-0.463	-0.379	-0.406		
	MOL-L	0.129	0.128	0.131	0.132			-0.456	-0.513	-0.364	-0.433		
12 − 2.4	INC	-5.5 × 10^− 2^	-6.9 × 10^− 2^	-4.0 × 10^− 2^	-3.4 × 10^− 2^			-0.320	-0.331	-0.279	-0.289		
	MOL-L	4.1 × 10^− 2^	2.6 × 10^− 2^	5.9 × 10^− 2^	6.5 × 10^− 2^			-0.326	-0.360	-0.263	-0.304		
12 − 2.8	INC	-5.0 × 10^− 2^	-6.0 × 10^− 2^	-3.8 × 10^− 2^	-3.4 × 10^− 2^			-0.339	-0.379	-0.271	-0.320		
	MOL-L	4.0 × 10^− 2^	2.9 × 10^− 2^	5.4 × 10^− 2^	5.9 × 10^− 2^			-0.327	-0.383	-0.246	-0.315		
16 − 2.0	INC	1.1 × 10^− 2^	1.7 × 10^− 2^	2.2 × 10^− 2^	1.8 × 10^− 2^	2.1 × 10^− 2^		-0.166	-0.178	-0.186	-0.146	-0.156	
	MOL-L	0.103	0.110	0.114	0.115	0.119		-0.184	-0.218	-0.240	-0.167	-0.194	
16 − 2.4	INC	3.0 × 10^− 2^	3.2 × 10^− 2^	3.2 × 10^− 2^	4.3 × 10^− 2^	4.6 × 10^− 2^		-0.122	-0.127	-0.127	-0.104	-0.109	
	MOL-L	0.126	0.128	0.127	0.143	0.147		-0.122	-0.149	-0.168	-0.108	-0.131	
16 − 2.8	INC	-4.2 × 10^− 2^	-3.3 × 10^− 2^	-2.6 × 10^− 2^	-3.2 × 10^− 2^	-3.0 × 10^− 2^		-3.7 × 10^− 2^	-3.6 × 10^− 2^	-3.5 × 10^− 2^	-2.6 × 10^− 2^	-2.6 × 10^− 2^	
	MOL-L	1.0 × 10^− 2^	1.8 × 10^− 2^	2.2 × 10^− 2^	2.4 × 10^− 2^	2.8 × 10^− 2^		-6.3 × 10^− 2^	-8.0 × 10^− 2^	-9.2 × 10^− 2^	-5.2 × 10^− 2^	-6.7 × 10^− 2^	
20 − 2.0	INC	-9.6 × 10^− 2^	-9.5 × 10^− 2^	-9.2 × 10^− 2^	-9.3 × 10^− 2^	-8.3 × 10^− 2^	-8.7 × 10^− 2^	-0.286	-0.315	-0.259	-0.277	-0.287	-0.246
	MOL-L	-2.6 × 10^− 2^	-2.8 × 10^− 2^	-1.9 × 10^− 2^	-1.9 × 10^− 2^	-9.7 × 10^− 3^	-1.1 × 10^− 2^	-0.306	-0.365	-0.274	-0.312	-0.331	-0.275
20 − 2.4	INC	-0.117	-0.117	-0.111	-0.110	-0.102	-0.106	-0.249	-0.281	-0.234	-0.243	-0.254	-0.216
	MOL-L	-4.7 × 10^− 2^	-4.9 × 10^− 2^	-3.8 × 10^− 2^	-3.6 × 10^− 2^	-2.9 × 10^− 2^	-2.9 × 10^− 2^	-0.261	-0.324	-0.231	-0.272	-0.293	-0.239
20 − 2.8	INC	-0.139	-0.144	-0.126	-0.123	-0.117	-0.112	-0.242	-0.266	-0.217	-0.232	-0.240	-0.204
	MOL-L	-5.7 × 10^− 2^	-6.4 × 10^− 2^	-4.2 × 10^− 2^	-3.8 × 10^− 2^	-3.2 × 10^− 2^	-2.5 × 10^− 2^	-0.246	-0.310	-0.216	-0.257	-0.278	-0.223

In practical scenarios, the fixed screws should not exhibit excessive displacement. However, under stress, the inevitability of screw movement or a tendency to move exists. Therefore, we favor forces that push inwards over those that tend to pull outwards. Our study results indicate that screws in commercial reconstruction plates are subject to outward pulling forces. In contrast, a rational design of customized reconstruction plates not only ensures that screws are subjected to inward pushing forces but also minimizes this value to a negligible impact. Comparing the commercial reconstruction plate and the 16 mm-2.8 mm customized reconstruction plates shows a clear gap.

### Finite element analysis results of the clinical application

The finite element analysis results were as follows. When simulated the stress condition of INC, the von Mises stress distributing on the 3D-printed customized reconstruction plate was between 0.24 MPa and 475.81 MPa which were within a safe range of the tensile yield strength of titanium as shown in Fig. 1E. When simulated the stress condition of L-MOL, the maximum von Mises stress was 423.21 MPa which was less than the tensile yield strength of titanium meaning strong and not easily broken as shown in Fig. 1F. In the shot, the Equivalent Stress was equally distributed in INC and L-MOL. For the bone tissues in the areas of rigid internal fixation as seen in Fig. 1G and H, the Equivalent Strain distribution was mostly less than 8 × 10^ − 4^ which meant the absorption of the bone because of the stress shielding effect. In addition, there may be bone degeneration at certain points. But both the stress shielding effect and the pathological had slight influences on the success of the operation.

## Discussion

For patients with segmental mandibular defects suffering from highly malignant tumors, facing a high rate of relapse or an inability to perform autologous bone grafts such as younger children or the old and weak, simple reconstruction plate repair surgeries could be a feasible transitional repair program [19, 20]. However, a large number of clinical retrospective studies have reported the exposure and fracture of the titanium plates, and screw loosening may occur from 3 months to 3 years after the operation, with a high incidence [21].

This project was to design a control experiment of customized reconstruction plates and commercial reconstruction plate for lateral segmental mandibular defect using finite element analysis and evaluate preliminarily how the shape parameters of the reconstruction plates determine the function of the reconstruction plates. From the mechanical analysis, we selected a group of parameters that can diminish the stress shielding effect and promote SBR. In all groups, the system of 16 mm-high, 2.8 mm-thick customized reconstruction plate with 10 screws was considered to be the most ideal design because of the most symmetrical and relatively small force. The most obvious finding of this study was that the design of reconstruction plates with better biomechanical properties can be obtained by adjusting the shape parameters. What’s more, a proper reconstruction plate can promote osteogenesis in children, although it did not completely avoid the stress shielding effect.

The insights gained from this study may be of assistance to the reconstruction of segmental mandibular defects. Vascularized bone flaps are still considered the gold-standard method for the majority of patients suffering from craniofacial bone deformities [22], besides, customized reconstruction plates bridging the bone stumps temporarily or permanently for patients who are too young or old to tolerate major surgical trauma or to be ineligible for autologous bone grafting are brought up as a desirable method [23, 24]. For inexperienced doctors, intraoperative bending of reconstruction plates is time-consuming, and repeated bending will lead to stress concentration in the metal and stress fatigue under the physiological loading^,^, leading to various complications such as fracture of titanium plates, corrosion, screw loosening, and bone resorption [25]. A controlled clinical study has found that reconstruction prebent plates according to the 3D-printed model of patients have better postoperative aesthetic and functional effects due to more accurate morphological fit [26]. In addition, it also had the advantages of reducing bone-plate spacing to reduce healing scars, reducing titanium plate exposure and stress fatigue, and protecting important anatomical structures such as tooth roots, blood vessels, and nerves [27]. Prospective clinical trials have shown that 3D-printed customized reconstruction implants with autogenous bone can be effectively used for head and neck reconstruction, simplifying surgical procedures and achieving high-precision jaw reconstruction, assuming that 3D-printed customized reconstruction implants are feasible, safe, and accurate [28]. Our research found that customized reconstruction plates designed and 3D printed from CT image data may have better biomechanical properties than pre-bent commercial reconstruction plates. Because the personalized reconstruction plate has a morphology almost completely fitted to the individual bone contour, it has a flexible shape design method of length, height, and thickness. These advantages were all confirmed by finite element studies.

The empirical findings in this study provide a new understanding of how the shape parameters such as height, thickness as well as the screws determine the biomechanical properties of the reconstruction plates. The results of this study indicate that on the premise of ensuring the strength of the reconstruction plates, the reconstruction plates can be designed to be smaller, thinner, and fit in shape. However, except for the obvious negative correlation between the maximum relative stress of the customized reconstruction plates and the height of them, the rest of the structural design values do not exhibit a clear positive or negative correlated relationship with the performance of the titanium plate, bone tissue, and screws. Interestingly, under the condition of a fixed titanium plate height, the maximum relative stress on the titanium plate initially increases and then decreases with the thickening of the plate. Moreover, the screws on the 16 mm-2.8 mm customized reconstruction plate are particularly excellent, and the reasons for this outcome require further investigation. These data suggest that a relatively optimal customized reconstruction plate design can be achieved through pre-finite element analysis by adjusting the shape parameters of the reconstruction plates to adapt to different bone densities, different bone defect types, and different occlusal conditions to achieve true personalization. In addition, finite element simulation occlusion analysis can find high-stress or high-strain points. On this basis, if the customized reconstruction plate is partially fine-adjusted, and the local position thickening or thinning in the overall structural design, a safer customized reconstruction plate with uniform stress distribution can be obtained. One can envision that, if we ascertain the principles for designing such customized reconstruction plates, and concurrently perform finite element prediction for personalized cases, then make structural micro-adjustments or topological optimization at stress concentration points or high-risk screw loosening sites, we would obtain an ideal reconstruction plate. This approach would obviate the need for autologous bone graft surgeries, significantly reducing surgery time, decreasing surgical trauma, and accelerating the postoperative healing process. Undoubtedly, this would transform future strategies in mandibular reconstruction.

While the results have interesting implications, any conclusions derived must be qualified by the limitations of the analysis procedure. First, the number and variety of shape parameters of a customized construction plate we studied were very basic and limited. Second, we have only considered the cases with the mandible under ordinary occlusal mandibular muscular forces. In the cases of the different large mandibular resections, the amount and patterns of the occlusal forces and the muscle forces in patients would be significantly different. Furthermore, it must be noted that the physiological changes over the course of bone healing are different because of differences in age, race, etc. In the future, we will focus on studying the behavior of plates and screws of different designs of customized reconstruction plates. For example, our study needs to include different types of mandibular defects, and even include the types of maxillary defects. With the difference in jaw defect types, the types of residual masticatory muscles and tooth occlusion will also change. Among them, the parameters of the jaw bone need to be considered for gender, age, and ethnic differences. In terms of customized reconstruction plate design, the parameters of the length, height, and thickness of the reconstruction plates need a classification design with a larger range and a smaller ladder gap to compare the quantitative or qualitative relationship between the parameters and the performance. In addition, the distribution of the screw holes needs special research, such as how to evenly distribute the holes in different areas, and the effect of the increase or decrease of the screw holes on the overall performance of the plates. Finally, we can also locally fine-tune the customized reconstruction plates through the finite element prediction method, which can be achieved by topological optimization or trying other ways. In conclusion, the design of the customized reconstruction plates should be as harmonious as possible with the mandible so that showing the biomechanical properties we are looking for.

## Conclusions

To our knowledge, many patients require mandibular reconstruction to maintain mandibular continuity but cannot tolerate autologous bone grafting. Retrospective studies are showing that showing that commercial plates have a high rate of postoperative complications such as fracture and exposure, and studies on reconstruction plate improvement are very limited. This study fills the gap in this line of research by utilizing three-dimensional finite element analysis.

3D-printed customized plates are considered to be suitable for use alone to reconstruct large bone defects in the mandible because of their good passive anatomical fitting, direct fixation without repeated bending, and adjustment of contour parameters to optimize biomechanical properties.

By the controlled trial and finite element analysis for the unilateral mandibular segmental defects reconstruction, we obtained a design criterion with some reference value, i.e., we can consider designing a customized reconstructive plate with a height of 16 mm, a thickness of 2.8 mm, and 10 screws for fixation. This design criterion was applied in the reconstructive surgery of one patient and achieved good surgical results as well as a satisfactory follow-up.

### Electronic supplementary material

Below is the link to the electronic supplementary material.

Supplementary Material 1

## Data Availability

The datasets used and/or analysed during the current study available from the corresponding author on reasonable request.

## Abbreviations

SBR: Spontaneous bone regeneration
CT: Computer tomography
3D: Three-dimensional
DICOM: Digital imaging and communication in medicine
STL: Stereolithographic
Hu: Hounsfield unit
NURBs: Non-uniform rational basis splines
FE: Finite element
INC: Incisal clenching
MOL-L: Left molar clenching
SM: Shallow masseter
DM: Deep masseter
AT: Anterior temporal muscle
MT: Medial temporal muscle
PT: Posterior temporal muscle
MP: Medial pterygoid
SLP: Superior lateral pterygoid
ILP: Inferior lateral pterygoid
