# Supplementary material for: Finite element analysis and clinical application of 3D-printed Ti alloy implant for the reconstruction of mandibular defects
Source: BMC Oral Health. 2024 Jan 17;24:95. doi: 10.1186/s12903-024-03857-y (PMC10792868; doi:10.1186/s12903-024-03857-y)
Supplement: Supplementary file 1 — Supplementary Material 1 [file 12903_2024_3857_MOESM1_ESM.doc]

Figure

Figure S1. The CT data processed by Mimics Medical software: (A) cortical bone; (B) cancellous bone; (C) teeth; (D) articular fossa.


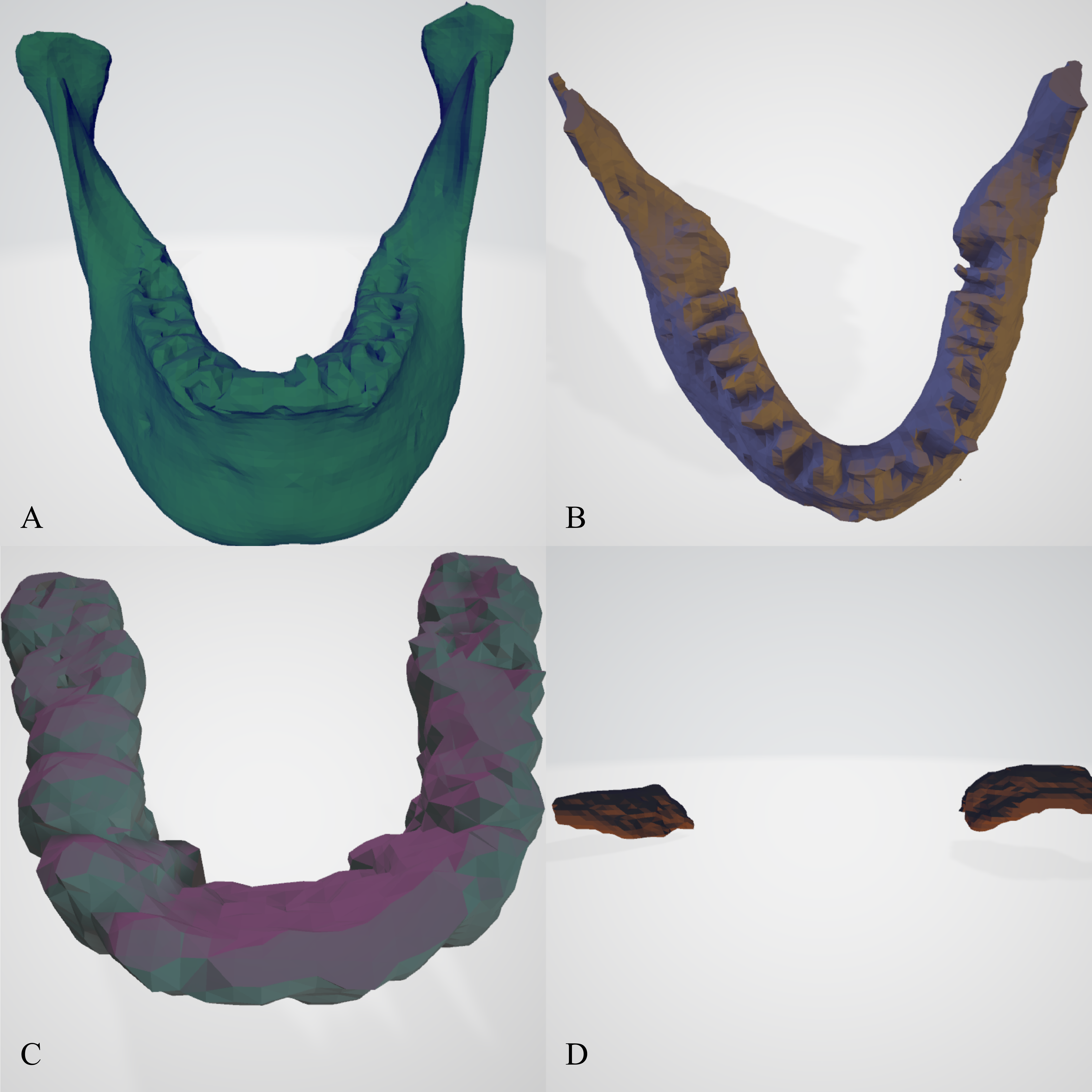


Figure S2. The STL data processed by Geomagic Wrap software and NX software: (A) cortical bone; (B) cancellous bone; (C) teeth; (D) articular fossa.


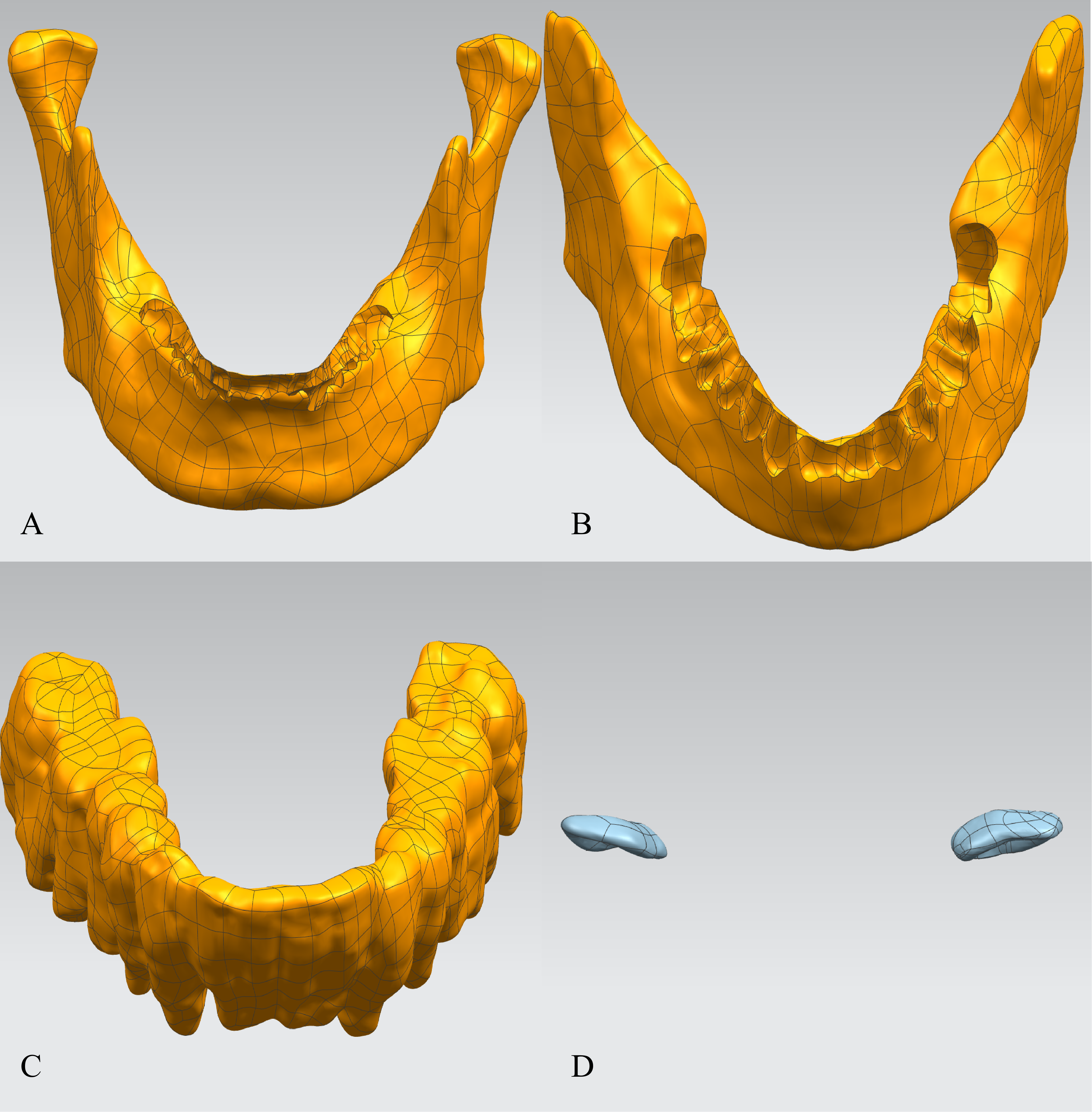


Figure S3. NX software was used to create 3D model of the mandible: (A) complete mandible + bilateral partial fossa; (B) mandibular segmental bone defect + bilateral partial articular fossa.


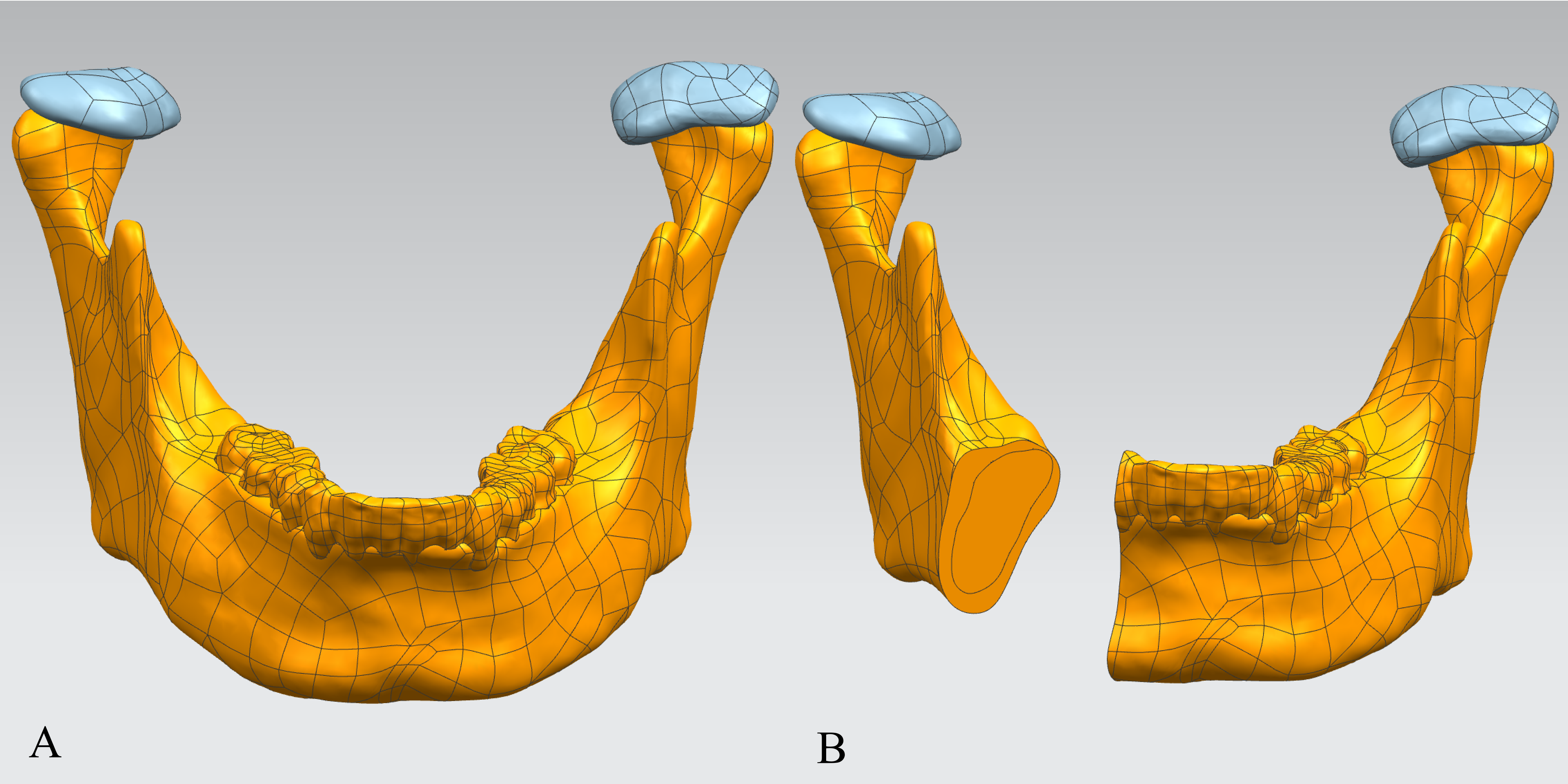


Figure S4.Create customized reconstruction board fitting surface body with Geomagic Design X software: (A) Create customized reconstruction board fitting surface body with domain-surface fitting function; (B) the front view; (C) the right side view; (D) the top view.


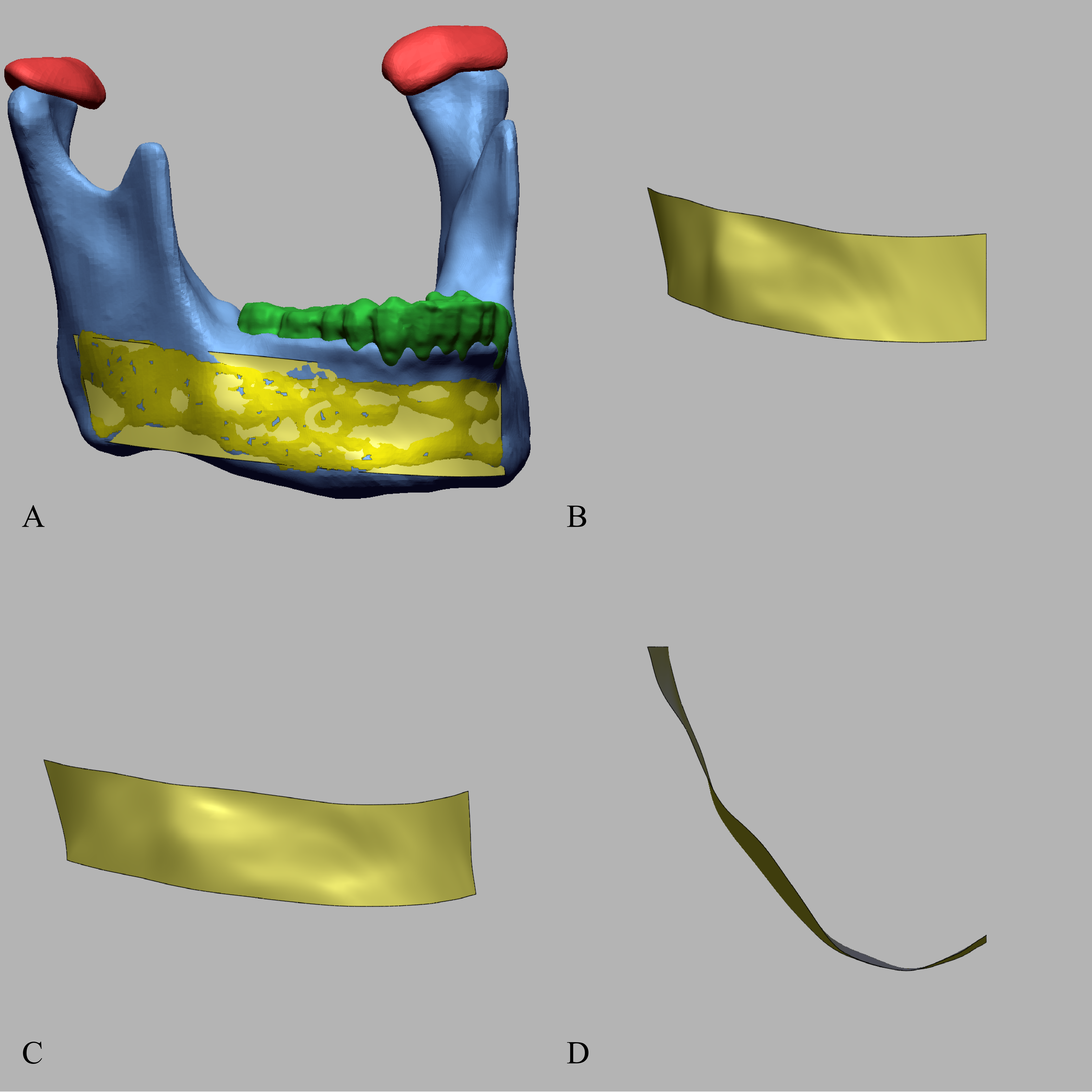


Figure S5.customized reconstruction plate: (A) 12mm high customized reconstruction plate, the right side view, left side view and top view; (B) 16mm highcustomized reconstruction plate, the right side view, left side view and top view; (C) 20mm high customized reconstruction plate, the right side view, left side view and top view.


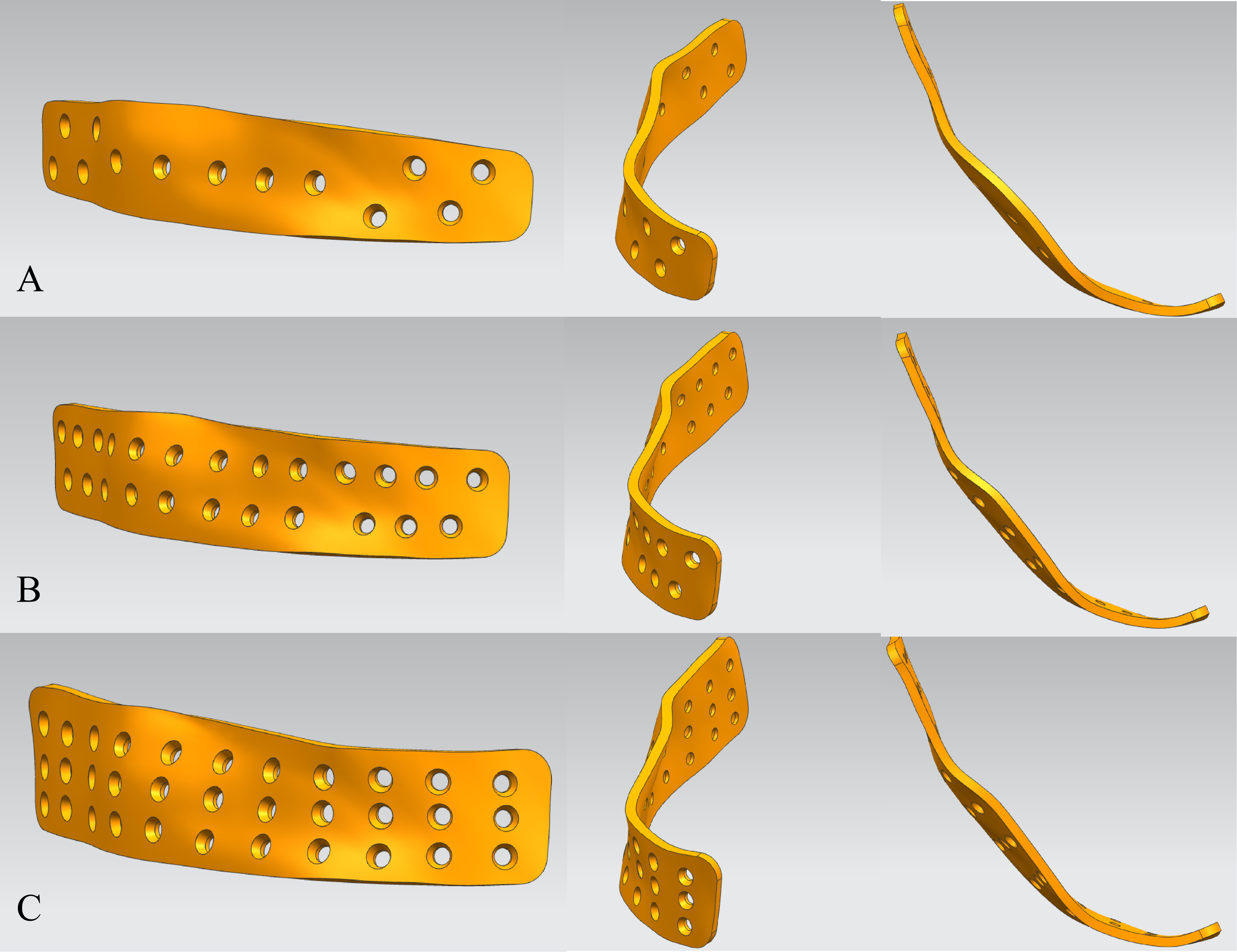


Figure S6.screw


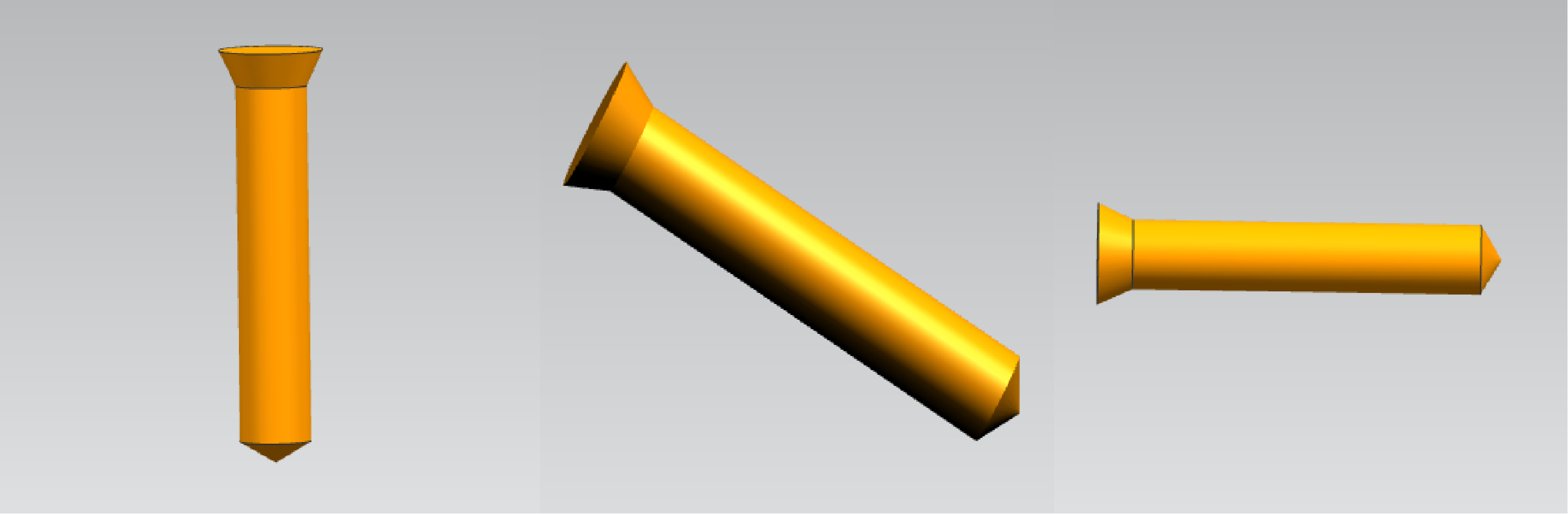


Figure S7. The right side view, left side view and top view of the commercial reconstruction plate.


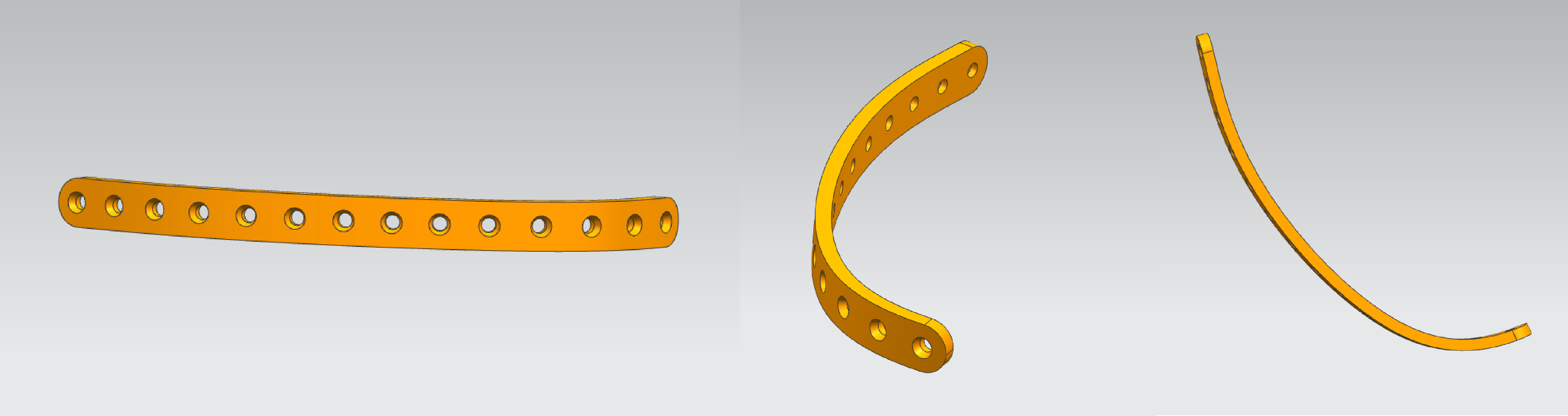


Figure S8. The screw distribution on the reconstruction plate. (A) commercial reconstruction plate. (B) 12mm-height reconstrction plate. (C) 16mm-height reconstrction plate. (D) 20mm-height reconstrction plate.

Figure S9. 3D finite element modeling of mandibular defects repaired by reconstruction plate: (A) repaired by 12mm high customized reconstruction plate; (B) repaired by 16mm high customized reconstruction plate; (C) repaired by 20mm high customized reconstruction plate; (D) repaired by commercial reconstruction plate.


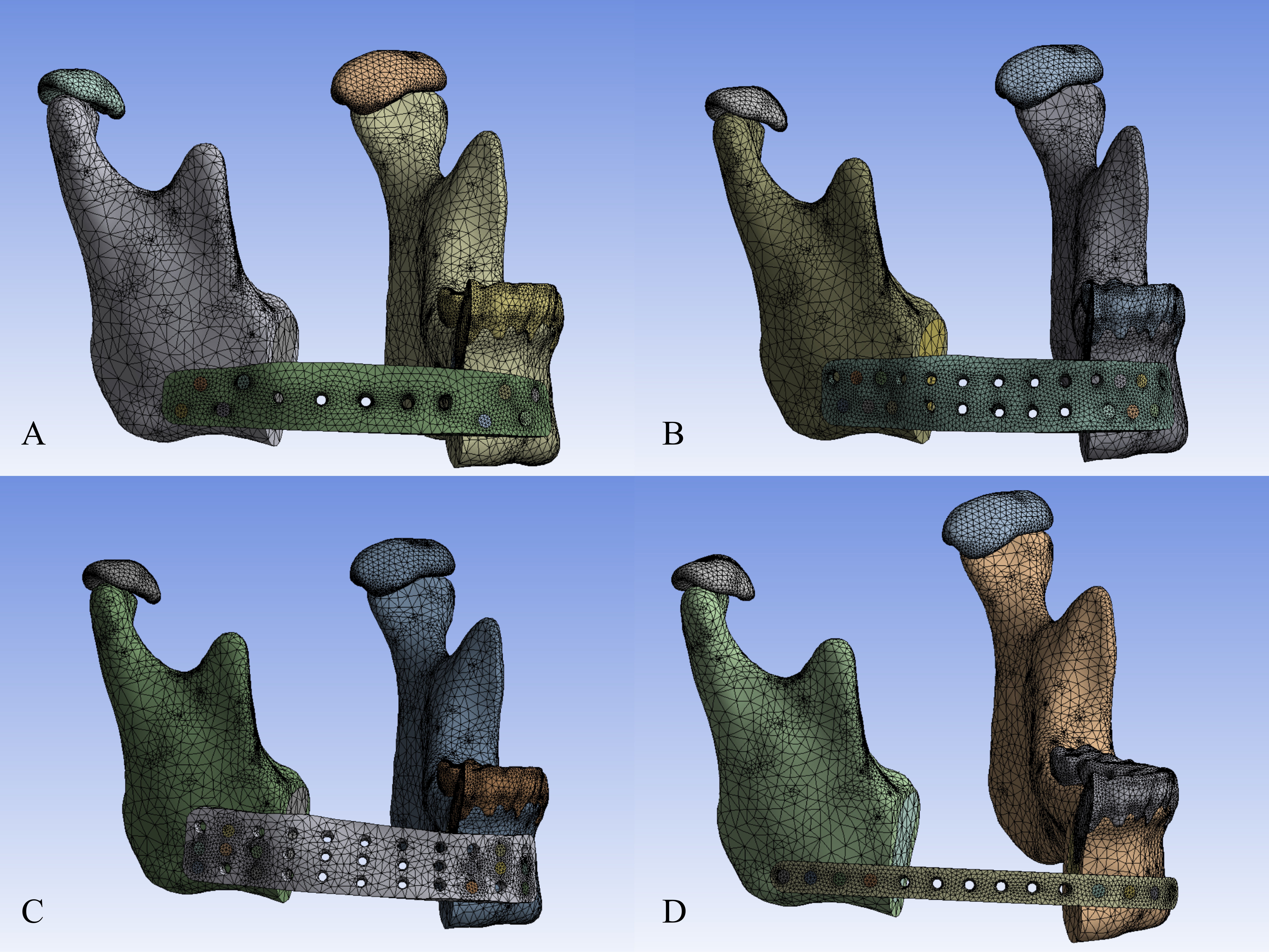


Figure S10. Supports and loads of a three-dimensional finite element model of mandibular defect under INC: (A), (B) fixed supports; (C)-(H ) occlusal force; (I),( J) masseter; (K) medial pterygoid; (L), (M), (N) temporalis; (O), (P) lateral pterygoid; Supports and loads of a three-dimensional finite element model of mandibular defect under MOL-L: (A)-(D) occlusal force; (E), (F) masseter; (G) medial pterygoid; (H), (I), (J) temporalis; (K) temporalis; (L), (M) fixed supports.


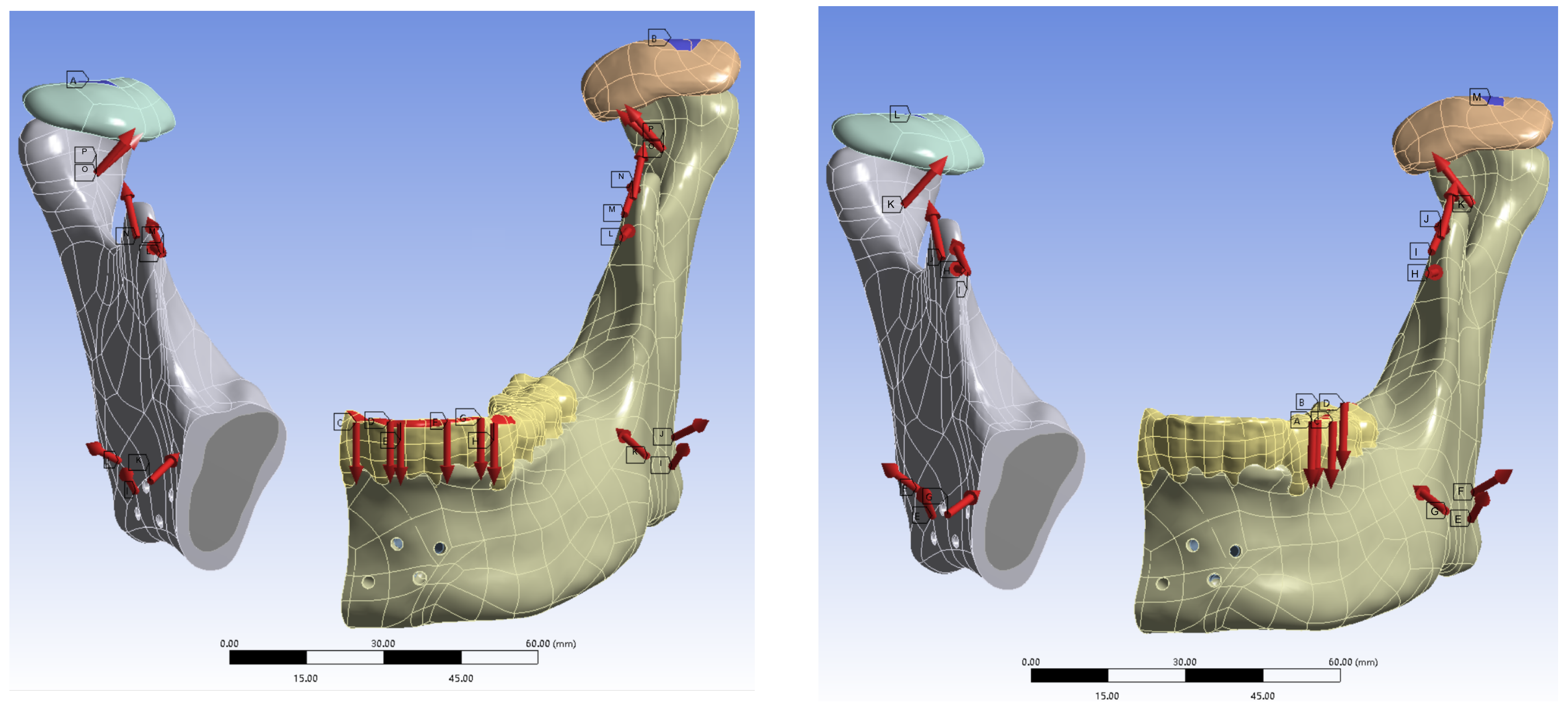


Figure S11. The four mechanical usage windows.


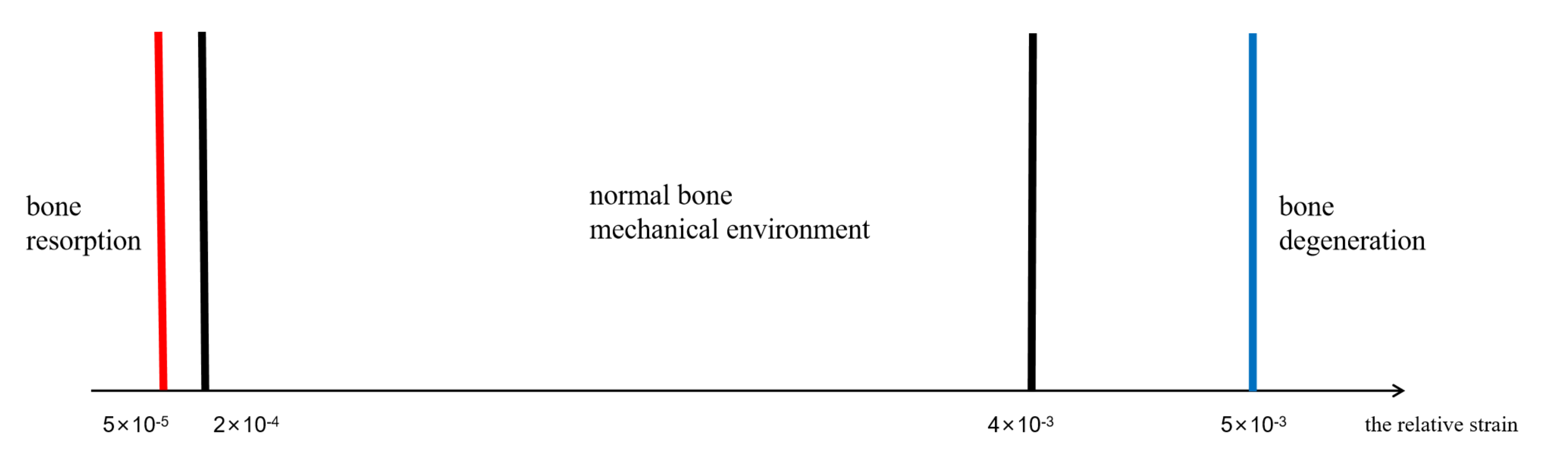


Table

Table S1. The design of the commercial reconstruction plate and the customized reconstruction plates

|  | **commercial reconstruction plate** | **customized reconstruction plates** | | | | | | | | |
| --- | --- | --- | --- | --- | --- | --- | --- | --- | --- | --- |
| Length/mm | 110 | 96 | 96 | 96 | 96 | 96 | 96 | 96 | 96 | 96 |
| Height/mm | 8 | 12 | 12 | 12 | 16 | 16 | 16 | 20 | 20 | 20 |
| Thickness/mm | 2.5 | 2.0 | 2.4 | 2.8 | 2.0 | 2.4 | 2.8 | 2.0 | 2.4 | 2.8 |
| Number of reserved holes | 14 | 13 | 13 | 13 | 24 | 24 | 24 | 33 | 33 | 33 |
| Number of fixing screws | 8 | 8 | 8 | 8 | 10 | 10 | 10 | 12 | 12 | 12 |
